# A network of transcriptomic signatures identifies novel comorbidity mechanisms between schizophrenia and somatic disorders

**DOI:** 10.1007/s44192-024-00063-8

**Published:** 2024-04-04

**Authors:** Youcheng Zhang, Vinay S. Bharadhwaj, Alpha T. Kodamullil, Carl Herrmann

**Affiliations:** 1 Institute of Pharmacy and Molecular Biotechnology (IPMB) & BioQuant, Universität Heidelberg, 69120 Heidelberg, Germany; 2 Department of Bioinformatics, Fraunhofer Institute for Algorithms and Scientific Computing (SCAI), 53757 Sankt Augustin, Germany

## Abstract

The clinical burden of mental illness, in particular schizophrenia and bipolar disorder, are driven by frequent chronic courses and increased mortality, as well as the risk for comorbid conditions such as cardiovascular disease and type 2 diabetes. Evidence suggests an overlap of molecular pathways between psychotic disorders and somatic comorbidities. In this study, we developed a computational framework to perform comorbidity modeling via an improved integrative unsupervised machine learning approach based on multi-rank non-negative matrix factorization (mrNMF). Using this procedure, we extracted molecular signatures potentially explaining shared comorbidity mechanisms. For this, 27 case–control microarray transcriptomic datasets across multiple tissues were collected, covering three main categories of conditions including psychotic disorders, cardiovascular diseases and type II diabetes. We addressed the limitation of normal NMF for parameter selection by introducing multi-rank ensembled NMF to identify signatures under various hierarchical levels simultaneously. Analysis of comorbidity signature pairs was performed to identify several potential mechanisms involving activation of inflammatory response auxiliarily interconnecting angiogenesis, oxidative response and GABAergic neuro-action. Overall, we proposed a general cross-cohorts computing workflow for investigating the comorbid pattern across multiple symptoms, applied it to the real-data comorbidity study on schizophrenia, and further discussed the potential for future application of the approach.

**Supplementary Information:**

The online version contains supplementary material available at 10.1007/s44192-024-00063-8.

## Introduction

Mental illnesses, in particular schizophrenia and bipolar disorder, are increasingly raising public health awareness globally. The clinical burden is not only driven by frequent chronic courses and increased mortality but also by the risk for somatic comorbid conditions such as cardiovascular disease [1] and type II diabetes [2]. People with mental illnesses often show an increased susceptibility to somatic illnesses. In fact, the comorbidity between psychotic diseases and somatic illnesses is mutual [3], with studies showing a higher risk of developing a psychiatric disorder for persons affected by certain somatic diseases [1, 3]. Conversely, the risk of somatic disease is approximately twice as high for persons with severe mental illnesses than for people without mental disorders [3]. Studies on genetic susceptibility have suggested a potential overlap of molecular mechanisms between psychotic disorders and somatic comorbidities [4, 5], such as shared gene risk loci that were demonstrated in several genotype studies [6], biomarkers that were dysregulated across psychosis and comorbid diseases in transcriptomic analysis [7, 8], and shared molecular pathways that were identified in integrated analysis [7].

To explore shared molecular mechanisms between somatic comorbidities and psychotic disorders, previous studies have been conducted with different approaches. For instance, genetic overlap analyses were performed on genome-wide summary statistics from two large-scale GWAS of schizophrenia and type II diabetes to discover the shared association between schizophrenia and type II diabetes [6]. Common differential expressed genes (DEGs) were analyzed as the comorbid genes across schizophrenia and type II diabetes and further identified the enriched gene ontology as well as transcription factors with these DEGs [9, 10]. Similarly, a set of susceptibility genes for schizophrenia and type II diabetes were retrieved respectively to identify the significant pathways crossing among two syndromes [10]. Mendelian randomization analysis was performed to establish the causal linkage of genetic variants associated with type II diabetes with the risk of schizophrenia using an inverse-variance weighted meta-analysis [11]. Multi-trait colocalization, which suggested a common genetic etiology between schizophrenia and cardiometabolic traits, was estimated with linkage disequilibrium score regression (LDSC), genetic covariance analyzer (GNOVA), and heritability estimation from summary statistics (ρ-HESS) [12], whereas polygenic risk scores were included additionally to identify the potential shared genetic variants and inferred the pathways involved [13]. The genetic-pleiotropy-informed method was introduced for improving gene discovery with the use of GWAS summary-statistics data to identify additional loci associated with schizophrenia, connecting various cardiovascular disease risk factors [14]. Molecular correlations and bi-directional Mendelian randomization (MR) were computed to assess the causality of an increased risk of cardiovascular disease in schizophrenia patients and to explore the underlying mediating factors [1]. Moreover, metagenes and molecular pattern discovery using independent component analysis (ICA) [15] in independent transcriptomes provided us new insights to study the molecular comorbidity [16–18].

However, due to the limited knowledge as well as the lack of systematic analysis on the comorbidity in psychotic disorders, the underlying mechanism remains to be explored. Therefore, in this study, we performed a systematic comorbidity analysis by developing a computational framework modeling leveraging a large-scale transcriptomic dataset via an improved integrative unsupervised machine learning approach based on multi-rank non-negative matrix factorization (mrNMF). Classical NMF allows decomposing the transcriptomic datasets into a fixed number of transcriptomic signatures (similar to meta-genes). However, this novel procedure allows to overcome the issue of determining the proper number of signatures present in a dataset, which is usually ambiguous. Hence, our approach allows to consider all possible decomposition ranks, extracting signatures at all granularities from broad signatures related to strong transcriptional differences down to fine-grained signatures representing less variance. Using this procedure, we extracted molecular signatures at various levels of granularity potentially explaining shared comorbid mechanisms. For this, 27 case–control microarray cohorts across multiple tissues were collected, covering three main categories of conditions including psychotic disorders, cardiovascular diseases, and type II diabetes. A reciprocal best-hit scoring matrix was computed to integrate signatures across different cohorts and to generate the disease signature graph while controlling confounding effects. Biological representation and gene analysis based on signature pairs of the graph was performed to identify comorbid processes and marker genes. GWAS mapping with the risk variants of each trait was implemented to validate the key genes in our analysis. Finally, we queried an independently curated knowledge graph to reveal the underlying relationships among shared molecular factors and the co-occurrence of these biological processes, further helping bridge the roadmap of the mechanism of the comorbidity.

## Method

### Datasets

27 case–control cohorts with 1163 individuals in total (633 patients and 530 healthy participants) were retrieved from Gene Expression Omnibus (GEO) repository, covering three main categories of conditions including psychotic disorders, cardiovascular diseases, and type II diabetes (See Online Resource 1, Supplementary Table S1). To reduce the system biases induced by different platforms, we only selected datasets using platform GPL570 [HG-U133_Plus_2] Affymetrix Human Genome U133 Plus 2.0 Array. Within the three main categories of conditions, various subtypes were collected: in the psychotic disorders category (PSY), schizophrenia (scz), bipolar disorders (bd), and major depression (mdd) were included, whereas for cardiovascular diseases (CVD), acute coronary syndrome (acs), coronary artery disease (cad), acute myocardial infarction (ami), peripheral arterial disease (pad), hypertension (hyp), and cardiomyopathy (cdm) were included. Categories are indicated in upper-case (PSY, CVD, T2D) while individual diseases are in lower-case. Each cohort involved individual samples from only one tissue, including brain, blood, islet, liver, etc. Other variables such as gender, age, BMI, etc., available in cohorts were extracted and stored in the metadata for further processing. Details were summarized in Online Resource 1, Supplementary Table S1.

### Data preprocessing

All the raw expression microarray data were retrieved from GEO as.CEL files. Frozen robust multiarray analysis (fRMA) [19, 20] was performed to preprocess raw data into expression data. RMA background correction, quantile normalization and robust weighted average summarization were applied to the fRMA preprocessing pipeline setting. Sample and outliers with missing values were removed, along with their corresponding expression data. Probes were mapped to gene symbols using biomaRt [21] package. Finally, we performed rowmean normalization (setting the mean value of each gene to 1).

### Multi-rank non-negative matrix factorization (mrNMF)

Multi-rank non-negative matrix factorization (mrNMF) is an improved version of non-negative matrix factorization (NMF), which is composed of four steps (1) signature identification at various factorization ranks using NMF (2) filtering of the signatures using a diagnosis association criterion graph building with reciprocal best hit scoring on signature pairs; (4) permutation testing and final signature graph generation.

Standard non-negative matrix factorization decomposes the data matrix (genes in rows and samples in columns) into two component matrices, gene-rank matrix (W) and rank-sample matrix (H). Here, the rank refers to the “signature” (column of the W matrix) which is a weighted genes vector, in which the weights are the exposure, representing the positive contributions of all genes to this signature. The value of the rank k is a tunable parameter, referring to the number of signatures. mrNMF used an ensemble strategy by applying a series of rank values from k = 2 to k = 20 on the decomposition procedure. Then all the resulting gene-signature W matrices and signature-sample H matrices were concatenated into one large W matrix and one large H matrix respectively. The loss function for each round of mrNMF with specific rank k is provided below.

$${min}_{WH}{||X-{W}_{(k)}{H}_{(k)}||}_{F}^{2}$$

$$s.t.W\ge 0,H\ge 0,k\ge 2$$

To identify the signatures that were significantly associated with the disease phenotype, a linear model was applied with values of each of the signatures in rank-sample H matrices as independent variables, group variable (case or control) as dependent variable and gender, age, and other available variables as covariates. A linear model with a t-test was performed to identify the signatures associated with the biological effect (P-value < 0.05). The corresponding column in the gene-rank W matrix and row in the rank-sample H matrix was kept while removing the non-significant signatures.

In the signature graph, edges between signatures were defined by measuring the similarity between two signatures. We assume that only a few genes significantly contribute to the signature, whereas the weight of most genes corresponds to random noise. Hence, we computed the Jaccard similarity coefficients using the top N (N = 1000) genes with top exposure values (Online Resource 1, Supplementary Fig. S1). We also compared the differences by using different values of N (Online Resource 1, Supplementary Fig. S2).

$$J(si{g}_{i},si{g}_{j})=\frac{|si{g}_{i}\cap si{g}_{j}|}{|si{g}_{i}\cup si{g}_{j}|}$$

We applied reciprocal best hit scoring to obtain the best concordance of pairs of signatures from different individual cohorts with Jaccard similarity coefficients as the scoring criterion.

To evaluate the empirical significance of the edges, a permutation procedure was implemented to identify the signature pairs that existed more than expected under a randomized case. The exposure values in gene-rank W matrices obtained in the mrNMF decomposition step were shuffled, followed by reciprocal best hit scoring and graph building. This procedure was repeated 20 times and the permutation test was computed on the occurrence of specific edges in the actual graph with unshuffled data on the permuted graph with shuffled data. Only significant edges with occurrence probability P ≤ 0.05 would be kept. In the end, the final signature graph is generated.

### Biological inference on signature pairs

Biological interpretation on the edges, in terms of enriched biological processes, was performed using the ClusterProfiler package [22] to perform enrichment analysis. For each edge, the shared genes in the signature pairs obtained from the previous step was used as the input. Gene Ontology resource (GO) from MSigDB [23, 24] was used. FDR correction was implemented to keep the significant GO terms (adjusted P-value < 0.05).

### Gene analysis on specific biological processes of signature pairs

Enriched genes in specific ontology terms (*acute inflammation*, *angiogenesis*, *oxidative stress,* and *GABAergic synapse*) were explored in detail. We first extracted the scz-t2d signature pairs that showed enrichment of one of these four ontology terms, meanwhile, we retrieved the enriched genes of these terms. For each gene and each signature pair, we obtained two exposure values, one from the schizophrenia signature, and the other from the comorbidity signature within the signature pairs. Genes with top 30 exposure value were ranked based on the geometric mean of the exposure values in the two individual signatures of the signature pair and visualized in a scatter plot with the exposure value for schizophrenia and the comorbid disease over the signature pairs as x and y, whereas we also annotated the types of genes retrieved from the previous section (*distinct, shared, schizophrenia specific, comorbid specific*).

### Mapping of the embedded genes in specific comorbid mechanism on the GWAS data

To validate the gene list identified in the scz-t2d comorbid signature pairs, we mapped the genes enriched in *acute inflammation*, *angiogenesis*, *oxidative process* and *GABAergic system* to the GWAS risk variants from schizophrenia and type 2 diabetes. 4988 associations in 142 studies with corresponding mapped gene information for schizophrenia and 5263 associations in 218 studies for type 2 diabetes were retrieved from GWAS catalog. The shared GWAS genes between schizophrenia and type 2 diabetes were treated as comorbid GWAS genes. The comorbid gene that we found in schizophrenia-t2d comorbid signature pairs were further summarized into different categories including “gwas_t2d”, “gwas_scz” and “gwas_shared”.

### Building the schizophrenia—type 2 diabetes comorbidity knowledge graph

We used a literature-curated knowledge-graph to build the connectivity paths from schizophrenia to type 2 diabetes and validate through an independent approach the specific processes identified from all the previous analyses in the study. Specific processes including *acute inflammation*, *angiogenesis*, *oxidative process,* and *GABAergic* were selected. We followed the procedure in the previous section to extract a list of query genes. The top 30 enriched genes with the highest exposure value of these processes were retrieved. With the selected processes and the corresponding high exposed genes as input, we fed them into a literature-curated knowledge graph database [25]. The graph with maximal connectivity depth = 3 (connected node degree) involved the specific processes and genes above was generated. Based on the graph, we created and visualized the subgraph for each biological process we wanted to highlight, by starting with the biological process and searching for all the shortest paths to schizophrenia node and type 2 diabetes node respectively.

## Result

### Overview of the SigGraph computation framework

**Fig. 1:**
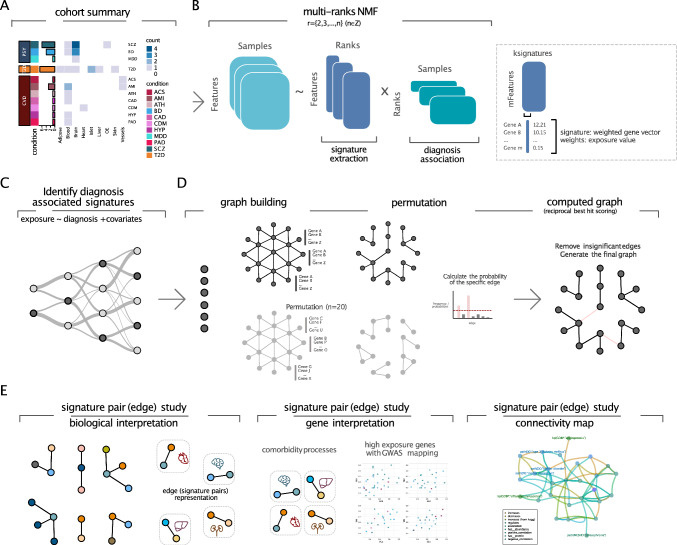
Overview of the signature graph computational framework based on multi-rank non-negative matrix factorization. **A** Descriptive summary of the transcriptomic cohorts, the number of cohorts by disease and tissue, scz (schizophrenia), bd (bipolar disorders), mdd (major depressive disorders), t2d (type 2 diabetes), acs (acute coronary syndrome), ami (acute myocardial infarction), ath (Atherosclerosis), cad (coronary artery disease), cdm (dilated cardiomyopathy), hyp (hypertension), pad (peripheral artery disease). **B** Algorithm of multi-rank non-negative matrix factorization. **C** Identification of diagnosis-associated signatures. **D** Graph building by reciprocal best hit matrices and permutation test. **E** Comorbidity analysis based on signature pairs including biological interpretation, high exposed gene identification and connectivity map establishment

To investigate the biological dimensions shared between psychotic disorders and somatic comorbidities, we developed a graph-based computational framework to perform comorbidity modeling via an improved integrative unsupervised machine learning approach based on multi-rank non-negative matrix factorization (mrNMF). Compared with the normal non-negative matrix factorization (NMF), mrNMF is optimized in several aspects. For instance, the factorization rank *k* must be provided in standard NMF, where a high rank might identify spurious signatures with little biological meaning whereas a low rank results in only a few signatures that might not be able to separate different biological factors in detail. Instead, we perform a multirank decomposition between *kmin* and *kmax*. Therefore, mrNMF can be regarded as an ensemble strategy that considers multiple ranks simultaneously to identify transcriptional signatures at various granularities. Moreover, with increasing rank parameter *k*, the biological signal captured by the parent nodes will split into their descendant nodes (Fig. 1), meaning that on different levels of *k*, signatures would capture different levels of the biological effects.

In the study, three large collections of transcriptomic datasets with 1163 individuals (633 patients and 530 healthy participants) were collected covering various psychotic disorders (schizophrenia, bipolar disorder, and major depressive disorder), type II diabetes and cardiovascular disease (coronary artery disease, peripheral artery disease, etc.) (Fig. 1, Supplementary Table S1). First, we applied mrNMF to decompose each preprocessed transcriptomic dataset independently (see Methods). The rank-sample *H* matrices were used to filter signatures (i.e. rows of the *H* matrix) significantly associated with diagnosis. Then, to infer correspondences between the gene signatures, we applied the reciprocal best-hit scoring as the similarity measurement metric on every pair of signatures obtained from different cohorts. As defined in the previous study [16, 18], a reciprocal best hit is found when a pair of signatures, each in a different cohort, is identified with each other as the best scoring match. In each signature pair, we focused on the top exposed genes and computed Jaccard similarity between the top 1000 genes to measure the similarity of the signature pairs. We have verified that the results are not dependent on this parameter (Online Resource 1, Supplementary Fig. S2). By doing so, we constructed a signature graph with edges weighted by Jaccard similarity between every best-matching signature pair. Third, to better understand the molecular origin of comorbidity, we performed an analysis on the graph components. Our hypothesis was that, when two connected signatures come from datasets corresponding to different diseases, the shared genes relate to common molecular processes found in both diseases. Thus, the comorbidity information can be extracted from the signature pair (edge) level. To remove random associations between signatures, we performed a permutation test by randomly constructing multiple graphs as controls and retained the signature pairs that occurred more than expected in the situation under randomization (see Methods). To identify the mechanism behind this, enriched biological processes were identified. Various types of comorbid genes were categorized and analyzed as well. Finally, to validate the results, GWAS data was used to validate the identified shared genes using genetic data.

### Description of the derived signature graph

**Fig. 2:**
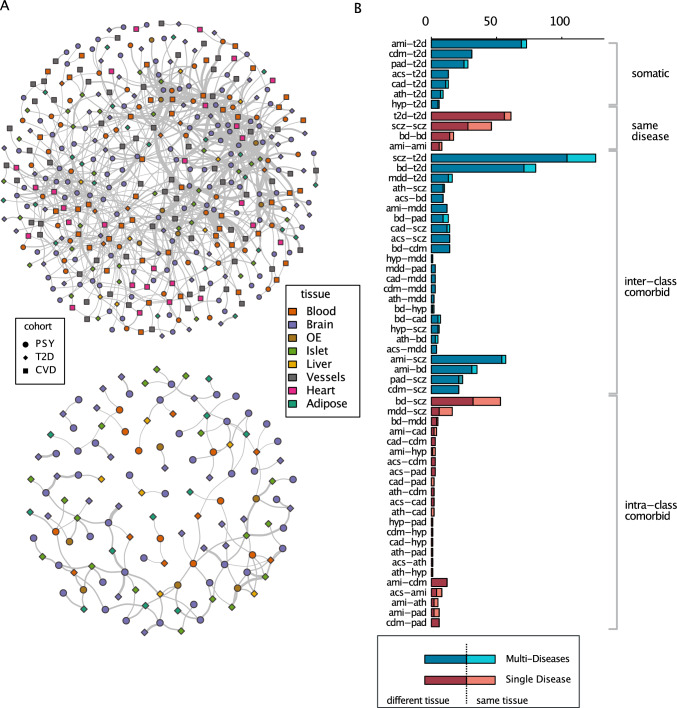
Computed signature graph and descriptive summary. **A** up Signature graph (full). Each node is an individual signature. Each edge indicates a reciprocal best hit matching between. Node shape represents disease class. Node color refers to the tissue origin. bottom Signature graph (scz-t2d). **B** Summary of the signature pairs (edges). Values in the x-axis represent the number of the signature pairs in specific categories. Single category refers to cohorts that only contain signatures from one of the disease categories among PSY, CVD and T2D. Comorbid category consists of multiple signatures from PSY-CVD, PSY-T2D and PSY + CVD + T2D at the same time. Somatic category is composed of CVD + T2D signatures

Our signature graph contains 419 signatures (represented as nodes) and 305 signature pairs (represented as edges) (Fig. 2A). These signatures have been identified at various factorization ranks, highlighting the relevance of our multi-rank strategy (Online Resource 1, Supplementary Fig. S4). These signatures belong to three different disease classes (PSY, T2D and CVD), 9 tissues and 58 types of edges (scz-scz, scz-t2d, bd-t2d, etc.). To standardize the notation, all the notations referring to disease classes (e.g. PSY, T2D, CVD) are in uppercase while those for specific diseases (scz, t2d, bd, ami, etc.) are in lowercase. We also extracted a subgraph containing only schizophrenia and type 2 diabetes signatures (Fig. 2A). The edges between individual signatures suggest shared biological processes leading to similar transcriptomic outcomes. Hence, information about shared molecular effects can be derived from a detailed analysis of signature pairs. Therefore, to explore the graph in detail, we summarized all the signature pairs (Fig. 2B). We categorized four different groups of signature pairs, namely *single* (signatures from the same disease, e.g. scz-scz), *intra-class comorbid* (signatures from the same disease class e.g. scz-bd), *inter-class comorbid* (signatures from distinct classes e.g. scz-t2d) and *somatic* (signatures from non-psychotic diseases). Among all signature pairs falling into the inter-class comorbid category, those connecting a psychiatric disorder and a somatic disease, in particular, schizophrenia—type 2 diabetes (scz-t2d), were observed most frequently. We questioned whether the tissue of origin represented a main confounder of the signature matching, in the sense that signatures derived from the same tissues would be associated more frequently. However, not only did the edges connect signatures from different diseases, they also frequently connected signatures obtained from different tissues. Therefore, to examine the tissue effect, we also summarized the number of cross-tissue signature pairs (Fig. 2B). It appears that signature pairs that linked individual signatures from different disease cohorts across different tissues were predominant, suggesting that tissue effect is not a main driver of signature clustering (Fig. 2B). Furthermore, within the signature pairs that belonged to the “multiple disease multiple tissue” category, a majority connected signatures issued from brain and blood, brain and islets and brain and vessel. Specifically, for schizophrenia—type 2 diabetes (scz-t2d) signature pairs, brain and islets, brain and adipose, and brain and liver were the major sources indicating again that there is no bias towards connecting signatures from the same tissue (Online Resource 1, Supplementary Fig. S3). To summarize, the computed signature graph displayed numerous connected signature pairs across cohorts and tissues, with a majority of connections between signatures extracted from schizophrenia datasets and type 2 diabetes datasets.

### Deciphering the variety of gene composition in comorbid signature pairs

**Fig. 3:**
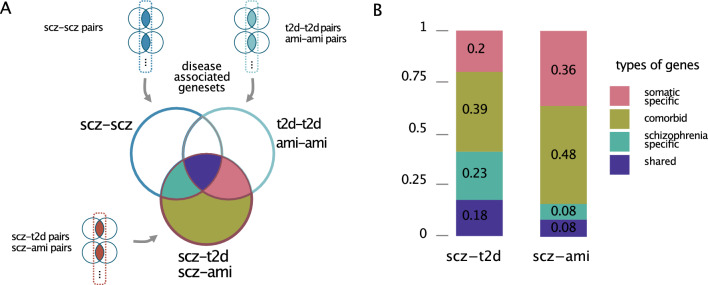
Identification of diverse types of comorbid signature pairs and the corresponding gene sets. **A** Illustration of categorizing types of genes in signature pairs, **B** Various types of comorbid genes in which the genes were labeled as “schizophrenia specific” in scz-t2d signature pairs that can only be found in the first single disease signature pair (e.g. scz-scz signature pairs), as “comorbidity specific” in scz-t2d signature pairs that can only be found in the first single disease signature pair (e.g. t2d-t2d or ami-ami signature pairs), as “shared” that can be found in the both scz-scz and t2d-t2d (or ami-ami) disease signature pair and “distinct” those that are not found in single disease signature pairs

Next, we wanted to understand to what extent the hybrid signatures connecting schizophrenia signatures to signatures from somatic diseases (called "comorbid pairs" in the remainder) contain specific biological signals that would not be identified by looking at each disease individually. We compared the union of all genes found associated to the comorbid signature pairs (i.e. scz-t2d or scz-ami) to the genes found in the schizophrenia pairs (scz-scz) and somatic pairs (t2d-t2d or ami-ami) and compared these gene sets. We labeled as *schizophrenia specific* the genes in the comorbid signature pairs that can only be found in the first single disease signature pair (e.g. scz-scz signature pairs), as *somatic specific* those that can only be found in the somatic disease (e.g. t2d-t2d or ami-ami), as *shared* those that can be found in both scz-scz and t2d-t2d or ami-ami disease signature pair and *comorbid* those that are not found in any of the single-disease signature pairs (Fig. 3A). Looking at two comorbidities with schizophrenia, diabetes and acute myocardial infarction, we observe that 39% (scz-t2d) and 48% (scz-ami) of the comorbid genes are indeed distinct from all the other categories, that is they were not previously identified in the single-disease signature pairs (scz-scz or t2d-t2d and scz-scz or ami-ami) and represent potential novel comorbid genes (Fig. 3B). These results indicate that the genes associated with comorbid signature pairs capture a specific biological signal that is distinct from the one explaining each disease separately.

### Elucidating roles of inflammatory response in schizophrenia—type 2 diabetes comorbidity

**Fig. 4:**
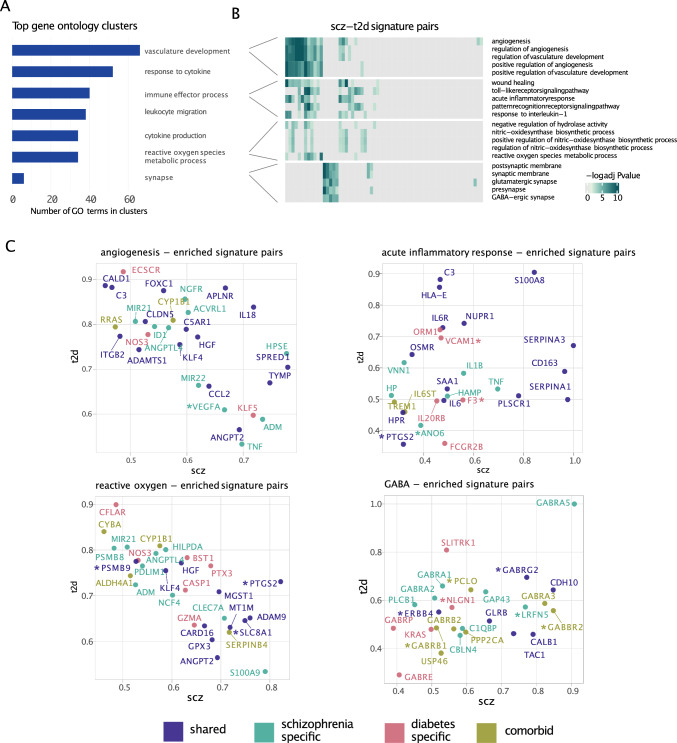
Biological representation of graph attributes. **A** Largest clusters with the most enriched processes in schizophrenia—type 2 diabetes signature pairs. **B** The top biological processes with most scz-t2d signature pairs enriched within three latest clusters vasculature development, immune effector process and ROS metabolic process and one minor cluster synapse. Each column represented one individual signature pair. Left annotations are the cluster names while the row names indicate the processes in each cluster. **C** Genes ranked by the exposure associated with the enriched pathways where x axis refers to the exposure of a gene in scz signature within a scz-t2d signature pair while y axis is the exposure of a gene in t2d signature within the signature pair. Colors refer to the type of genes which corresponds to the classification of various types of genes in Fig. 3B. GWAS risk variants were marked with asterisks

In the remainder of this study, we decided to focus on the comorbidity between schizophrenia and diabetes, as these two disorders showed the highest number of connections in the signature graph (Fig. 2B). To explore the underlying mechanisms linked to comorbidity, we conducted a fine-grained functional enrichment analysis on each of the scz-t2d signature pairs using the overlapping gene lists as input (see Method). 590 enriched GO terms were identified, where many of these terms were found to be enriched across multiple signature pairs, which indicates the robustness of this enrichment (full sheet in Online Resource 2). After clustering these terms, we considered the largest clusters grouping most GO terms (Fig. 4A). We identified numerous terms covering a wide range of activities of immune processes, which involved the production, activation, migration and regulation of immune cells, and production of cytokines (Fig. 4A). This finding indicates that immunological processes and inflammatory responses play an important role in the shared molecular dimension between schizophrenia and diabetes. This is consistent with numerous studies on possible comorbidity mechanisms, confirming the validity of our strategy [12, 26]. We also found less expected clusters such as one containing terms related to vasculature development. For example, the term angiogenesis was identified as being enriched in 19 scz-t2d pairs. It has been previously reported that regulation of the microvascular environment leads to ​​vascular abnormalities inducing angiogenic factors and neuroinflammation in schizophrenia patients [27–29]. Vascular abnormalities were also described in the pathogenesis of diabetes involving diabetic neuropathy such as changes of microvascular environment [30, 31]. Besides, *reactive oxygen metabolism* and *oxidase process* were also observed to be enriched in comorbid signature pairs that are interdependent with the inflammation. These impaired processes have been mentioned in several previous studies on t2d comorbidity of schizophrenia where oxidative-antioxidant imbalance led to the dysregulation of lipid transportation which caused and deteriorated the diabetes syndrome [29, 32]. In addition, other processes also raised our interest though they were found in a smaller cluster. For instance, we observed one cluster annotated with synapse-related processes, e.g. *GABA-ergic synapse* was found to be enriched in 5 signature pairs. It has been reported that GABA-ergic system components also closely interact with pro-inflammatory factors in the dys-regulation of immune signaling pathways [33]. In addition, processes related to the dopamine pathway were found to be enriched in eight scz-t2d pairs involving four different schizophrenia cohorts and three diabetes datasets (Online Resource 1, supplementary table S3 and Online Resource 2).

In summary, our analysis of comorbid pairs highlights known biological processes related to shared by both diseases, as inflammatory processes, but reveals additional intriguing pathways related to angiogenesis and GABAergic synapses.

### Investigating genes in key comorbid mechanism across schizophrenia and type 2 diabetes

To understand what specific genes were identified in the biological processes, we extracted the gene exposure from the signatures in the comorbid signature pairs (see Method, Fig. 4B and Online Resource 1, Supplementary Fig. S1). We focussed on the three representative processes in the largest cluster respectively including angiogenesis, acute inflammatory response and ROS, and one in small but highly related with previous terms, GABAergic synapse. All the scz-t2d comorbid signature pairs enriched for these terms were retrieved and the top responsive genes with the highest exposure were ranked (Fig. 4C and Method). For angiogenesis-enriched signature pairs, we observed highly exposed genes in both scz and t2d signatures, e.g. *IL18, APLNR, NGFR, TYMP, SPRED1* and *HPSE*. Some of these genes including *IL18, APLNR, NGFR* and *SPRED1* were already shown to be dysregulated in various processes such as inflammation, energy metabolism, peripheral neuropathy and cell plasticity in both diseases [34–42]. For instance, *APLNR,* encoding apelin APJ system is implicated in psychosis and neuropathy processes in chronic schizophrenia [42, 43] but also acts as a promising biomarker for the treatment of type 2 diabetes given its functions in energy metabolism in insulin resistance progress via adipokines involvement [44]. Other genes were not yet described in the context of comorbidities. Specifically, *TYMP,* which promotes angiogenesis and stimulates the growth of endothelial cells, was already found associated with endothelial dysfunction and induced diabetic-like symptoms in studies of type 2 diabetes [45], but has not been described in the context of schizophrenia. However, mitochondrial neurogastrointestinal dysfunction, associated with biallelic variants in the *TYMP* gene, was observed commonly in schizophrenia [46, 47]. We also investigated genes related to the enriched GABAergic system processes. Here, *GABRA5* was identified in scz-t2d comorbid genes. This gene is involved in neurotransmitter regulation in psychosis [48]. In diabetic patients, the GABAergic system regulates GABA release in the endocrine system which induces membrane depolarization and increases insulin secretion [48]. It is also associated with episodic memory dysfunction and lower cognitive performance [49]. Other genes such as *CDH10,* encoding neuronal cell–adhesion molecules, were already shown to be associated with neuropsychiatric disorders (e.g. autism spectrum disorders)[50] but not specifically to schizophrenia so far. In diabetes, *CDH10* and the cadherin family were identified in several complications of diabetes mellitus such as diabetic peripheral neuropathy [50, 51] and diabetic kidney disease [50–52].

Comparing these genes with genes associated with GWAS variants, we found 33 common genes (genes marked with asterisk in Fig. 4C). For example, *VEGFA* marked in angiogenesis is a risk variant specifically in t2d and has also been identified as an important biomarker regulating energy metabolism and vascular blood flow in schizophrenia [53]. PSMB9 which is related to immunoproteasome, was found in oxidative-related processes [57]. ERBB4 is implicated in schizophrenia patients as one of the key dysregulated signaling in the development of cortical inhibitory GABAergic circuits indicated in GABA-enriched signature pairs (Fig. 4C) [54].

To summarize, our analysis of comorbid signature pairs connecting schizophrenia and diabetes highlighted several enriched functional terms, among which acute inflammatory response, angiogenesis, oxidative process and GABAergic pathway. We noticed that most of the identified processes were to some extent associated with inflammatory response, therefore suggesting inflammation as a central mechanism in schizophrenia—type 2 diabetes comorbidity. Many of these processes had been connected separately to schizophrenia and diabetes in the literature, but our analysis strengthens the hypothesis that these processes represent shared molecular processes explaining the higher susceptibility to diabetes for schizophrenia patients.

### Building the potential scz-t2d comorbidity mechanism through knowledge graphs

**Fig. 5:**
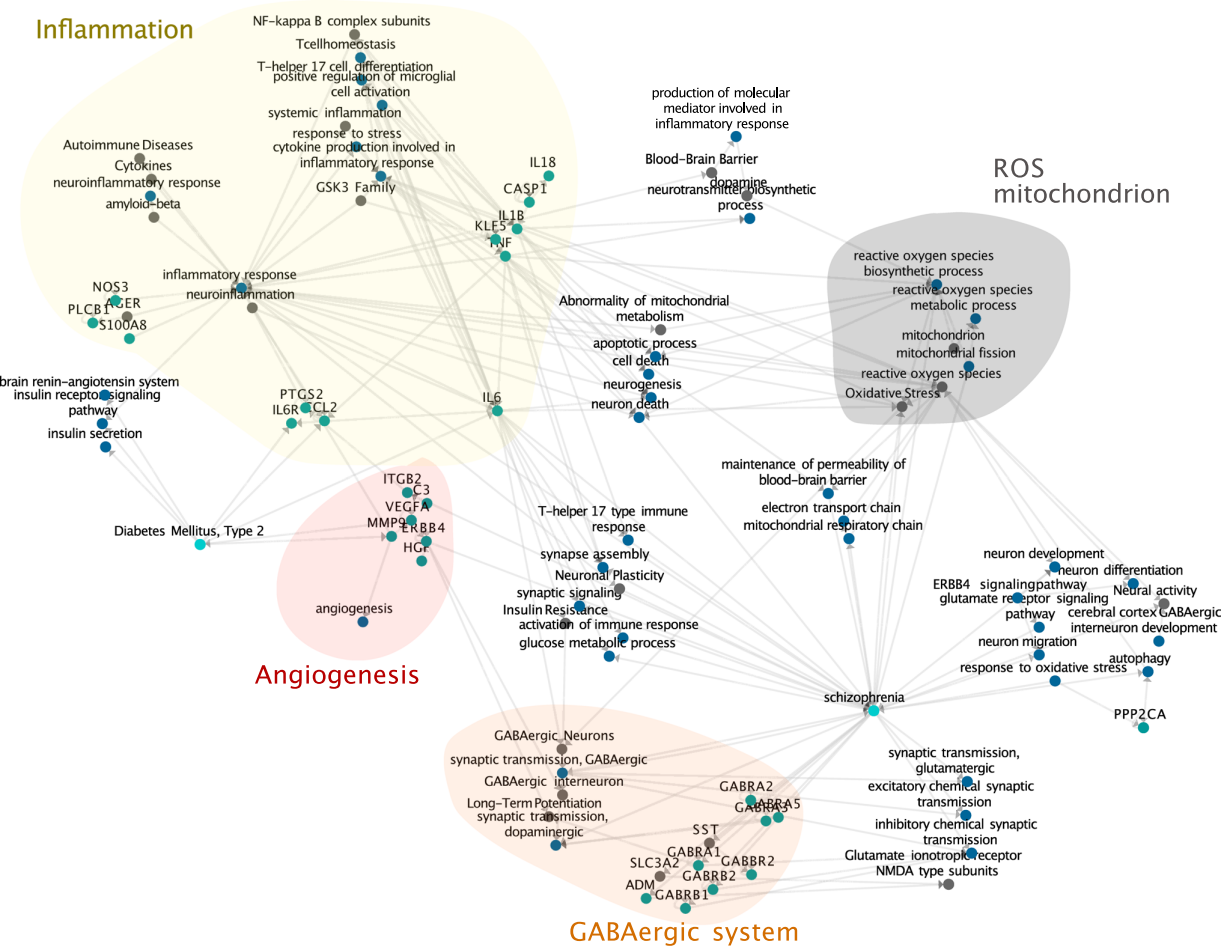
Schizophrenia—type 2 diabetes comorbidity knowledge-graph. Specific processes selected from all the previous analysis in the study including angiogenesis, inflammatory response, response to oxidative stress and GABA-ergic synapse, mapped on a knowledge-graph extracted from a literature-curated database. The output was generated and contained curated-connected nodes with maximal depth = 5 of these specific processes. Each node represents a specific entity (e.g. HGNC genes, GO biological processes, etc.) while each edge refers to a relationship or interaction between the connected nodes

To summarize the potential mechanism underlying the comorbidity, a schizophrenia—type 2 diabetes comorbidity map was built using a disease knowledge graph specifically curated for schizophrenia, bipolar disorder and type 2 diabetes (See Method). We centered the analysis on the four processes (ROS, angiogenesis, acute inflammatory response and GABAergic processes), with a central focus on the inflammatory response, and identified the connecting path between the diseases, intending to illustrate the specific roles of the processes in connecting schizophrenia and type 2 diabetes. Finally, we identified the subgraphs with strong evidence for each process separately by extracting the paths connecting schizophrenia and type 2 diabetes (Fig. 5).

In this map, inflammation, as well as other related terms (neuroinflammation, systemic inflammation, etc.), appears as a highly connected hub node, with connections to the insulin receptor signaling pathway and hence to diabetes, as well as multiple connections, in particular through *IL6*, to schizophrenia. *IL6* and its receptor, *IL6R*, appear to directly connect diabetes to schizophrenia, with multiple connections bridging through the other highlighted processes including GABAergic neurons, oxidative stress, and neuronal plasticity. Molecular factors such as *TNF* and *IL6*, related to inflammatory response, have also been identified as top exposure genes in our previous analysis (Fig. 4C).

Reactive oxygen species process played its roles via regulating the mitochondrial respiratory chain, electron transport chain, apoptotic process, in particular neuron death, and further processes related to the regulation of the blood–brain barrier, with multiple connections to schizophrenia. The connection to diabetes leads again through *IL6*. The angiogenesis term is connected to central genes such as *MMP9* and *VEGFA*, which establish the connection to the GABAergic system. Finally, terms in the knowledge graph related to the GABAergic system, as well as multiple associated genes are found to display numerous direct connections to schizophrenia, as well as to insulin resistance, confirming the role of GABAergic deregulation in the etiology of diabetes [48].

Based on the knowledge graph, we identified additional processes related to neural development (neuronal plasticity, neuronal signal transduction), energy metabolism (energy reserve metabolic process, mitochondrion, abnormality of mitochondrial metabolism), immune response (neuroinflammatory response, activation of immune response), blood–brain systems (blood–brain barrier, brain renin-angiotensin system), insulin system (insulin secretion, insulin receptor signaling) and cell death (apoptotic process, neuron death). By combining these finding obtained from the knowledge graph and the enrichment analysis, we noticed that the co-occurrence of these processes forms a complex mechanism connecting not only oxidative response, cell death, but also other reactions such as cell self-protection and cell recovery, strengthening the hypothesis that the comorbidity of type 2 diabetes in schizophrenia could be inflammatory processes triggering damage and repair processes.

## Discussion

In summary, we presented a computational framework to perform comorbidity modeling via an improved integrative unsupervised machine learning approach based on multi-rank non-negative matrix factorization (mrNMF), based on the analysis of a large number of disease related transcriptomic datasets. Our approach addresses the parameter selection limitation of normal NMF by introducing multi-rank ensembled NMF to comprehensively identify biological signals from signatures under different rank. Using a robust network based approach, we have identified modules of connected disease signatures. A large number of these connections related signatures from distinct diseases, highlighting the shared transcriptomic dimension between different diseases. We have focused our analysis on the relationship between schizophrenia and diabetes, and highlighted significant central roles of the immune system and inflammatory response, as well as its close interaction with angiogenesis, reactive-oxygen related processes and GABAergic system in shared transcriptomic signatures.

One limitation of the study is that the overlapping pathways were identified when disease-associated pathways co-occurred in both diseases. Hence, we cannot distinguish between causative effects, leading to real comorbidity risk, and shared downstream consequences of each disease. However, our GWAS analysis appears to confirm several genes identified by our unsupervised analysis as shared risk variants in diabetes and schizophrenia, such as *ERBB4* and *NLGN1*. *NLGN1* is an interesting hit, for its role in the interaction of endothelial cells with extracellular matrix during angiogenesis and its downregulation in diabetic patients [55]. At the same time, variants of the paralogous *NLGN2* gene have been identified as risk variants in schizophrenia due to their role as cell-adhesion molecules in post-synaptic membrane [56]. This variant is associated with loss-of-function in the formation of GABAergic synapses, connecting back to one of the key processes identified in our analysis. Furthermore, we also identified terms related to the dopaminergic system including the synthesis, secretion, transport and metabolism of dopamine in our scz-t2d comorbidity modeling (Online Resource 1, supplementary table S3 and Online Resource 2). Interestingly, despite its putative involvement in the pathogenesis of schizophrenia, we did not identify enrichment of terms related to the serotonergic system in the scz-t2d pairs. Nevertheless, the investigation on the role of serotonin in schizophrenia from different studies tends to show inconsistent results [58]. In contrast to the relationship between serotonergic systems and schizophrenia, the evidence for the associations between serotonin and major depressive disorder are relatively more solid [59] and we did identify the serotonin transport, secretion, and signaling in the comorbid analysis of major depressive disorder (Online Resource 1, supplementary table S4).

Our study highlighting shared molecular pathways might have clinical implications in the treatment of patients with comorbidities. Molecular markers identified in comorbidity modeling might be translated to biomarkers in clinical use for diagnosing schizophrenia patients with and without diabetes or metabolic syndrome. Moreover, it could contribute to the identification of the molecular factors that contribute to side effects of the antipsychotic medications [60], and could lead to further development of the next generation antipsychotic drug taking into account the potential adversarial effects on comorbid conditions.

Overall, the computational framework based on mrNMF signature graph is a powerful and unbiased approach for cross-cohort modeling of disease comorbidity. By means of ensembling multi-ranks, it captures transcriptomic signatures at different levels of granularity, providing a complete description of cellular processes that might underlie disease comorbidities.

### Supplementary Information

Below is the link to the electronic supplementary material.

Supplementary file1 (PDF 364 KB)

Supplementary file2 (XLSX 276 KB)

## Data Availability

All data set used in this study is publicly available, and retrieved from Gene Expression Omnibus (GEO). Details for accessing the used data is provided within Online Resource 1, Supplementary Table S1.

## Code availability

All analyses in this study are implemented in R 4.1.0. The codes are available from the corresponding author on reasonable request.

## References

[1] Veeneman RR, Vermeulen JM, Abdellaoui A, Sanderson E, Wootton RE, Tadros R (2022). Exploring the relationship between schizophrenia and cardiovascular disease: a genetic correlation and multivariable mendelian randomization study. Schizophr Bull.

[2] Lin PI, Shuldiner AR (2010). Rethinking the genetic basis for comorbidity of schizophrenia and type 2 diabetes. Schizophr Res.

[3] Dornquast C, Tomzik J, Reinhold T, Walle M, Mönter N, Berghöfer A (2017). To what extent are psychiatrists aware of the comorbid somatic illnesses of their patients with serious mental illnesses?—a cross-sectional secondary data analysis. BMC Health Serv Res.

[4] Postolache TT, Del Bosque-Plata L, Jabbour S, Vergare M, Wu R, Gragnoli C (2019). Co-shared genetics and possible risk gene pathway partially explain the comorbidity of schizophrenia, major depressive disorder, type 2 diabetes, and metabolic syndrome. Am J Med Genet B Neuropsychiatr Genet.

[5] Strawbridge RJ, Johnston KJA, Bailey MES, Baldassarre D, Cullen B, Eriksson P (2021). The overlap of genetic susceptibility to schizophrenia and cardiometabolic disease can be used to identify metabolically different groups of individuals. Sci Rep.

[6] Hackinger S, Prins B, Mamakou V, Zengini E, Marouli E, Brčić L (2018). Evidence for genetic contribution to the increased risk of type 2 diabetes in schizophrenia. Transl Psychiatry.

[7] Liu H, Sun Y, Zhang X, Li S, Hu D, Xiao L (2020). Integrated analysis of summary statistics to identify pleiotropic genes and pathways for the comorbidity of schizophrenia and cardiometabolic disease. Front Psychiatry.

[8] Mizuki Y, Sakamoto S, Okahisa Y, Yada Y, Hashimoto N, Takaki M (2021). Mechanisms underlying the comorbidity of schizophrenia and type 2 diabetes mellitus. Int J Neuropsychopharmacol.

[9] Rahman MR, Islam T, Nicoletti F, Petralia MC, Ciurleo R, Fisicaro F (2021). Identification of common pathogenetic processes between schizophrenia and diabetes mellitus by systems biology analysis. Genes.

[10] Liu Y, Li Z, Zhang M, Deng Y, Yi Z, Shi T (2013). Exploring the pathogenetic association between schizophrenia and type 2 diabetes mellitus diseases based on pathway analysis. BMC Med Genom.

[11] Li Z, Chen P, Chen J, Xu Y, Wang Q, Li X (2018). Glucose and insulin-related traits, type 2 diabetes and risk of schizophrenia: a mendelian randomization study. EBioMedicine.

[12] Perry BI, Bowker N, Burgess S, Wareham NJ, Upthegrove R, Jones PB (2022). Evidence for shared genetic aetiology between schizophrenia, cardiometabolic, and inflammation-related traits: genetic correlation and colocalization analyses. Schizophr Bull Open.

[13] So H-C, Chau K-L, Ao F-K, Mo C-H, Sham P-C (2019). Exploring shared genetic bases and causal relationships of schizophrenia and bipolar disorder with 28 cardiovascular and metabolic traits. Psychol Med.

[14] Andreassen OA, Djurovic S, Thompson WK, Schork AJ, Kendler KS, O’Donovan MC (2013). Improved detection of common variants associated with schizophrenia by leveraging pleiotropy with cardiovascular-disease risk factors. Am J Hum Genet.

[15] Jutten C, Herault J (1991). Blind separation of sources, part I: an adaptive algorithm based on neuromimetic architecture. Signal Process.

[16] Cantini L, Kairov U, de Reyniès A, Barillot E, Radvanyi F, Zinovyev A (2019). Assessing reproducibility of matrix factorization methods in independent transcriptomes. Bioinformatics.

[17] Brunet J-P, Tamayo P, Golub TR, Mesirov JP (2004). Metagenes and molecular pattern discovery using matrix factorization. Proc Natl Acad Sci U S A.

[18] Greco A, Sanchez Valle J, Pancaldi V, Baudot A, Barillot E, Caselle M (2019). Molecular inverse comorbidity between alzheimer’s disease and lung cancer: new insights from matrix factorization. Int J Mol Sci.

[19] McCall MN, Bolstad BM, Irizarry RA (2010). Frozen robust multiarray analysis (fRMA). Biostatistics.

[20] McCall MN, Jaffee HA, Irizarry RA (2012). fRMA ST: frozen robust multiarray analysis for Affymetrix Exon and Gene ST arrays. Bioinformatics.

[21] Durinck S, Spellman PT, Birney E, Huber W (2009). Mapping identifiers for the integration of genomic datasets with the R/Bioconductor package biomaRt. Nat Protoc.

[22] Wu T, Hu E, Xu S, Chen M, Guo P, Dai Z (2021). clusterProfiler 4.0: a universal enrichment tool for interpreting omics data. Innovation.

[23] Subramanian A, Tamayo P, Mootha VK, Mukherjee S, Ebert BL, Gillette MA (2005). Gene set enrichment analysis: a knowledge-based approach for interpreting genome-wide expression profiles. Proc Natl Acad Sci U S A.

[24] Liberzon A, Birger C, Thorvaldsdóttir H, Ghandi M, Mesirov JP, Tamayo P (2015). The Molecular signatures database (MSigDB) hallmark gene set collection. Cell Syst.

[25] Bharadhwaj VS, Mubeen S, Sargsyan A, Jose GM, Geissler S, Hofmann-Apitius M (2023). Integrative analysis to identify shared mechanisms between schizophrenia and bipolar disorder and their comorbidities. Prog Neuropsychopharmacol Biol Psychiatry.

[26] Perry BI, Burgess S, Jones HJ, Zammit S, Upthegrove R, Mason AM (2021). The potential shared role of inflammation in insulin resistance and schizophrenia: a bidirectional two-sample mendelian randomization study. PLoS Med.

[27] Casas BS, Vitória G, Prieto CP, Casas M, Chacón C, Uhrig M (2022). Schizophrenia-derived hiPSC brain microvascular endothelial-like cells show impairments in angiogenesis and blood–brain barrier function. Mol Psychiatry.

[28] Najjar S, Pahlajani S, De Sanctis V, Stern JNH, Najjar A, Chong D (2017). Neurovascular unit dysfunction and blood-brain barrier hyperpermeability contribute to schizophrenia neurobiology: a theoretical integration of clinical and experimental evidence. Front Psychiatry.

[29] Schmidt-Kastner R, van Os J, Esquivel G, Steinbusch HWM, Rutten BPF (2012). An environmental analysis of genes associated with schizophrenia: hypoxia and vascular factors as interacting elements in the neurodevelopmental model. Mol Psychiatr.

[30] Tahergorabi Z, Khazaei M (2012). Imbalance of angiogenesis in diabetic complications: the mechanisms. Int J Prev Med.

[31] Fadini GP, Albiero M, Bonora BM, Avogaro A (2019). Angiogenic abnormalities in diabetes mellitus: mechanistic and clinical aspects. J Clin Endocrinol Metab.

[32] Bryll A, Skrzypek J, Krzyściak W, Szelągowska M, Śmierciak N, Kozicz T (2020). Oxidative-Antioxidant imbalance and impaired glucose metabolism in schizophrenia. Biomolecules.

[33] Shan Y, Zhao J, Zheng Y, Guo S, Schrodi SJ, He D (2023). Understanding the function of the GABAergic system and its potential role in rheumatoid arthritis. Front Immunol.

[34] Li R, Wang B, Wu C, Li D, Wu Y, Ye L (2021). Acidic fibroblast growth factor attenuates type 2 diabetes-induced demyelination via suppressing oxidative stress damage. Cell Death Dis.

[35] Zakharyan R, Atshemyan S, Gevorgyan A, Boyajyan A (2014). Nerve growth factor and its receptor in schizophrenia. BBA Clinical.

[36] Messiaen L, Yao S, Brems H, Callens T, Sathienkijkanchai A, Denayer E (2009). Clinical and mutational spectrum of neurofibromatosis type 1–like syndrome. JAMA.

[37] Meng S, Cao JT, Zhang B, Zhou Q, Shen CX, Wang CQ (2012). Downregulation of microRNA-126 in endothelial progenitor cells from diabetes patients, impairs their functional properties, via target gene Spred-1. J Mol Cell Cardiol.

[38] Zaharieva E, Kamenov Z, Velikova T, Tsakova A, El-Darawish Y, Okamura H (2018). Interleukin-18 serum level is elevated in type 2 diabetes and latent autoimmune diabetes. Endocr Connect.

[39] Syed AAS, He L, Shi Y, Mahmood S (2021). Elevated levels of IL-18 associated with schizophrenia and first episode psychosis: a systematic review and meta-analysis. Early Interv Psychiatry.

[40] Moreno M, Lanni A (2016). Hormonal and neuroendocrine regulation of energy balance.

[41] ChapmanNigel A, DupréDenis J, RaineyJan K (2014). The apelin receptor: physiology, pathology, cell signalling, and ligand modulation of a peptide-activated class A GPCR1. Biochem Cell Biol.

[42] Lv S-Y, Chen W-D, Wang Y-D (2020). The apelin/APJ system in psychosis and neuropathy. Front Pharmacol.

[43] Sahpolat M, Ari M, Kokacya MH (2020). Plasma apelin, visfatin and resistin levels in patients with first episode psychosis and chronic schizophrenia. Clin Psychopharmacol Neurosci.

[44] Hu H, He L, Li L, Chen L (2016). Apelin/APJ system as a therapeutic target in diabetes and its complications. Mol Genet Metab.

[45] Marei I, Chidiac O, Thomas B, Pasquier J, Dargham S, Robay A (2022). Angiogenic content of microparticles in patients with diabetes and coronary artery disease predicts networks of endothelial dysfunction. Cardiovasc Diabetol.

[46] Colijn MA (2022). The co-occurrence of gastrointestinal symptoms and psychosis: diagnostic considerations. Prim Care Companion CNS Disord.

[47] Pacitti D, Levene M, Garone C, Nirmalananthan N, Bax BE (2018). Mitochondrial Neurogastrointestinal encephalomyopathy: into the fourth decade, what we have learned so far. Front Genet.

[48] Wan Y, Wang Q, Prud’homme GJ (2015). GABAergic system in the endocrine pancreas: a new target for diabetes treatment. Diabetes Metab Syndr Obes.

[49] Fernández-Matarrubia M, Matías-Guiu JA, Cabrera-Martín MN, Moreno-Ramos T, Valles-Salgado M, Carreras JL (2017). Episodic memory dysfunction in behavioral variant frontotemporal dementia: a clinical and FDG-PET study. J Alzheimers Dis.

[50] Anney RJL (2013). Common genetic variants in autism spectrum disorders. The neuroscience of autism spectrum disorders.

[51] Gu Y, Qiu Z-L, Liu D-Z, Sun G-L, Guan Y-C, Hei Z-Q (2018). Differential gene expression profiling of the sciatic nerve in type 1 and type 2 diabetic mice. Biomed Rep.

[52] Tao Y, Wei X, Yue Y, Wang J, Li J, Shen L (2021). Extracellular vesicle-derived AEBP1 mRNA as a novel candidate biomarker for diabetic kidney disease. J Transl Med.

[53] Pillai A, Howell KR, Ahmed AO, Weinberg D, Allen KM, Bruggemann J (2016). Association of serum VEGF levels with prefrontal cortex volume in schizophrenia. Mol Psychiatry.

[54] Luykx JJ, Vinkers CH, Bakker SC, Visser WF, van Boxmeer L, Strengman E (2012). A common variant in ERBB4 regulates GABA concentrations in human cerebrospinal fluid. Neuropsychopharmacology.

[55] Yang C, Eleftheriadou M, Kelaini S, Morrison T, González MV, Caines R (2020). Targeting QKI-7 in vivo restores endothelial cell function in diabetes. Nat Commun.

[56] Sun C, Cheng M-C, Qin R, Liao D-L, Chen T-T, Koong F-J (2011). Identification and functional characterization of rare mutations of the neuroligin-2 gene (NLGN2) associated with schizophrenia. Hum Mol Genet.

[57] Kim M-J, Serwa RA, Samluk Ł, Suppanz I, Kodroń A, Stępkowski TM (2023). Immunoproteasome-specific subunit PSMB9 induction is required to regulate cellular proteostasis upon mitochondrial dysfunction. Nat Commun.

[58] Quednow BB, Geyer MA, Halberstadt AL, Müller CP, Cunningham KA (2020). Chapter 39 Serotonin and schizophrenia. Handbook of the Behavioral Neurobiology of Serotonin.

[59] Moncrieff J, Cooper RE, Stockmann T, Amendola S, Hengartner MP, Horowitz MA (2022). The serotonin theory of depression: a systematic umbrella review of the evidence. Mol Psychiatry.

[60] Annamalai A, Tek C (2015). An overview of diabetes management in schizophrenia patients: office based strategies for primary care practitioners and endocrinologists. Int J Endocrinol.

