# Supplementary material for: A network of transcriptomic signatures identifies novel comorbidity mechanisms between schizophrenia and somatic disorders
Source: Discov Ment Health. 2024 Apr 4;4(1):11. doi: 10.1007/s44192-024-00063-8 (PMC10994898; doi:10.1007/s44192-024-00063-8)
Supplement: Supplementary file 1 — Supplementary file1 (PDF 364 KB) [file 44192_2024_63_MOESM1_ESM.pdf]

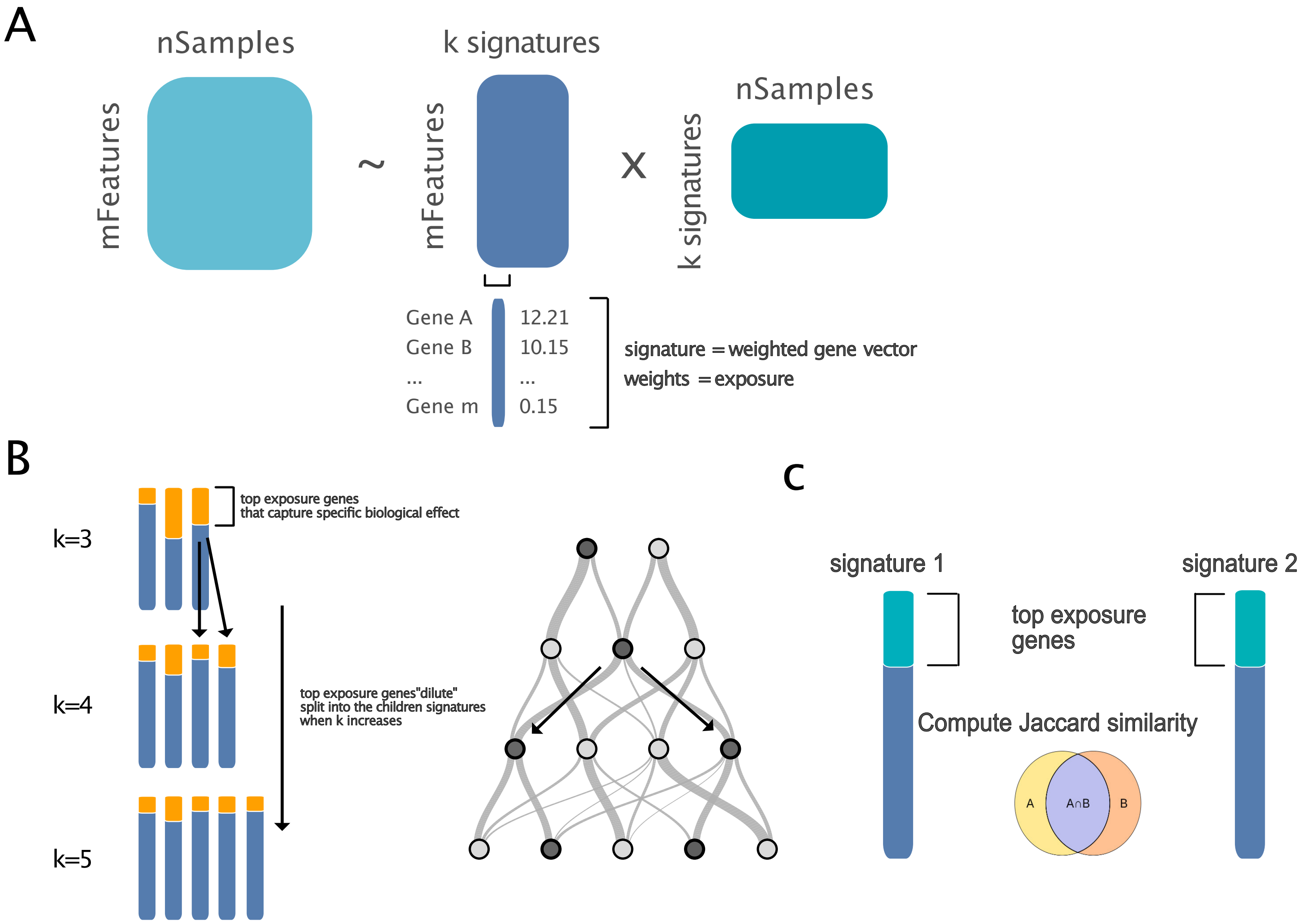

Supplementary Fig. S1 A Illustration of the non-negative matrix factorization and the matrix outputs B left Matrix decomposition with a series of different rank parameters k from k = 2 to k = 20 right Riverplot of the matrix factorization with a series of different rank where each node refers to signature, width of the flow refers to the similarity of the signatures and colors were annotated to indicate the diagnosis association of the signatures C Jaccard similarity calculated by selected top N of the genes from the signatures

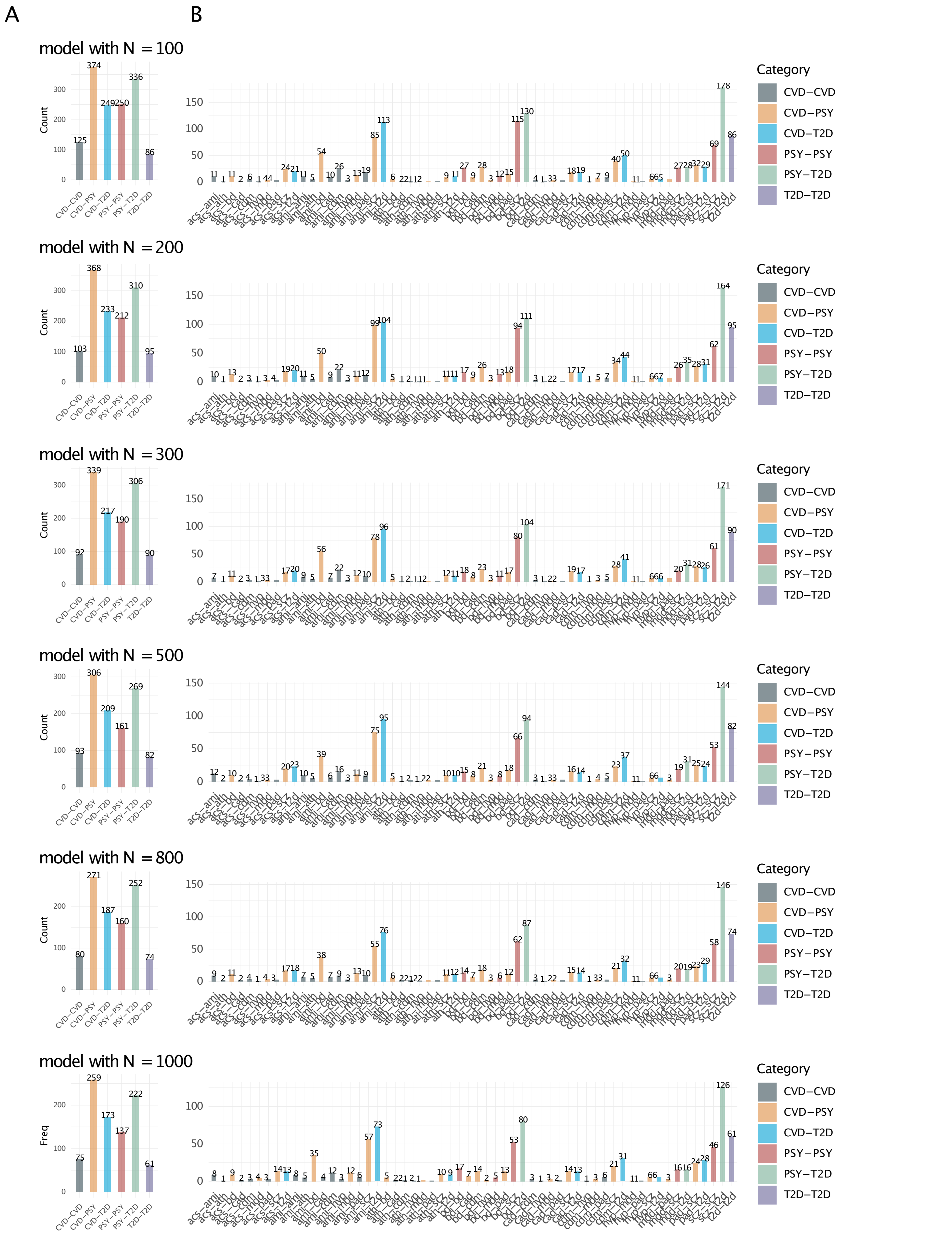

Supplementary Fig. S2 Summary of the identified signature pairs based on models with different top N genes A Counts of identified signature pairs based on different models by the disease major class (e.g. PSY, T2D and CVD) B Counts of identified signature pairs based on different models by the condition class (e.g. scz, t2d, ami, etc..)

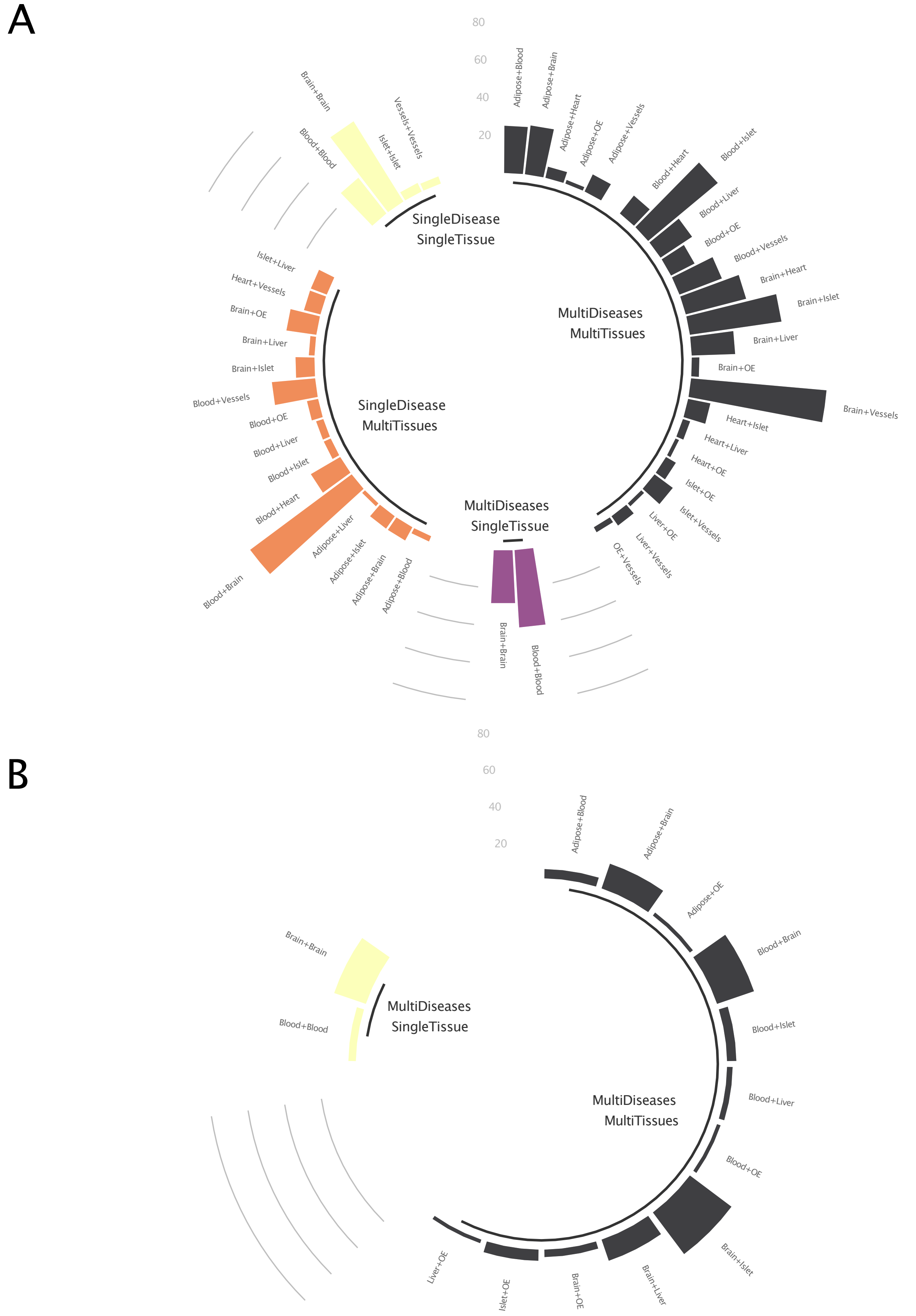

Supplementary Fig. S3 A Summary of the tissue–tissue combination of all the signature pairs
B Summary of the tissue–tissue combination of the schizophrenia –type 2 diabetes signature pairs

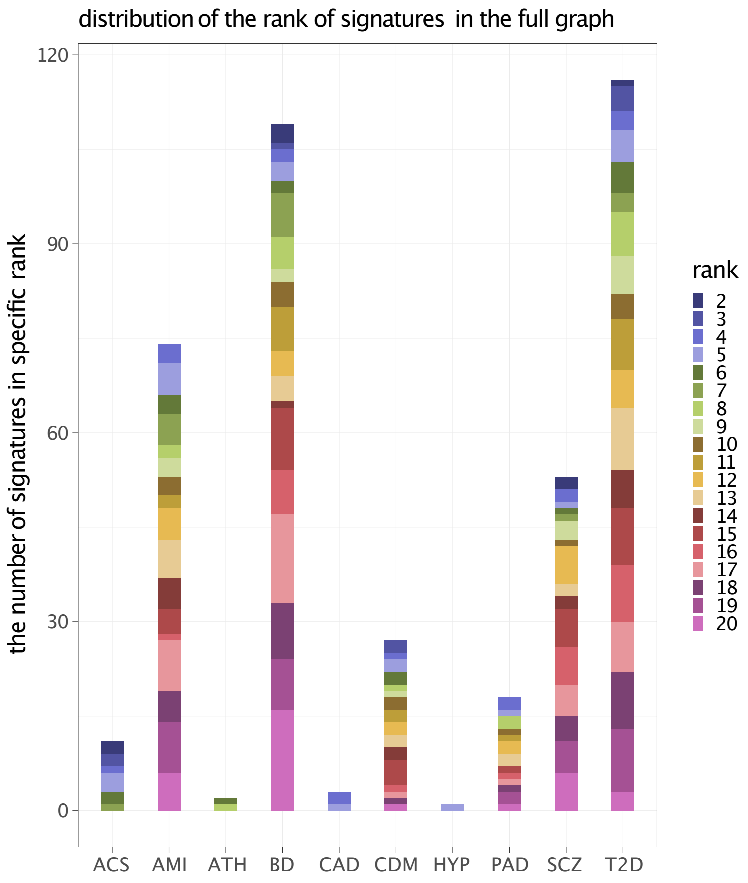

Supplementary Fig. 4 Tracking signatures in the full graph identified from different ranks in the decomposition model

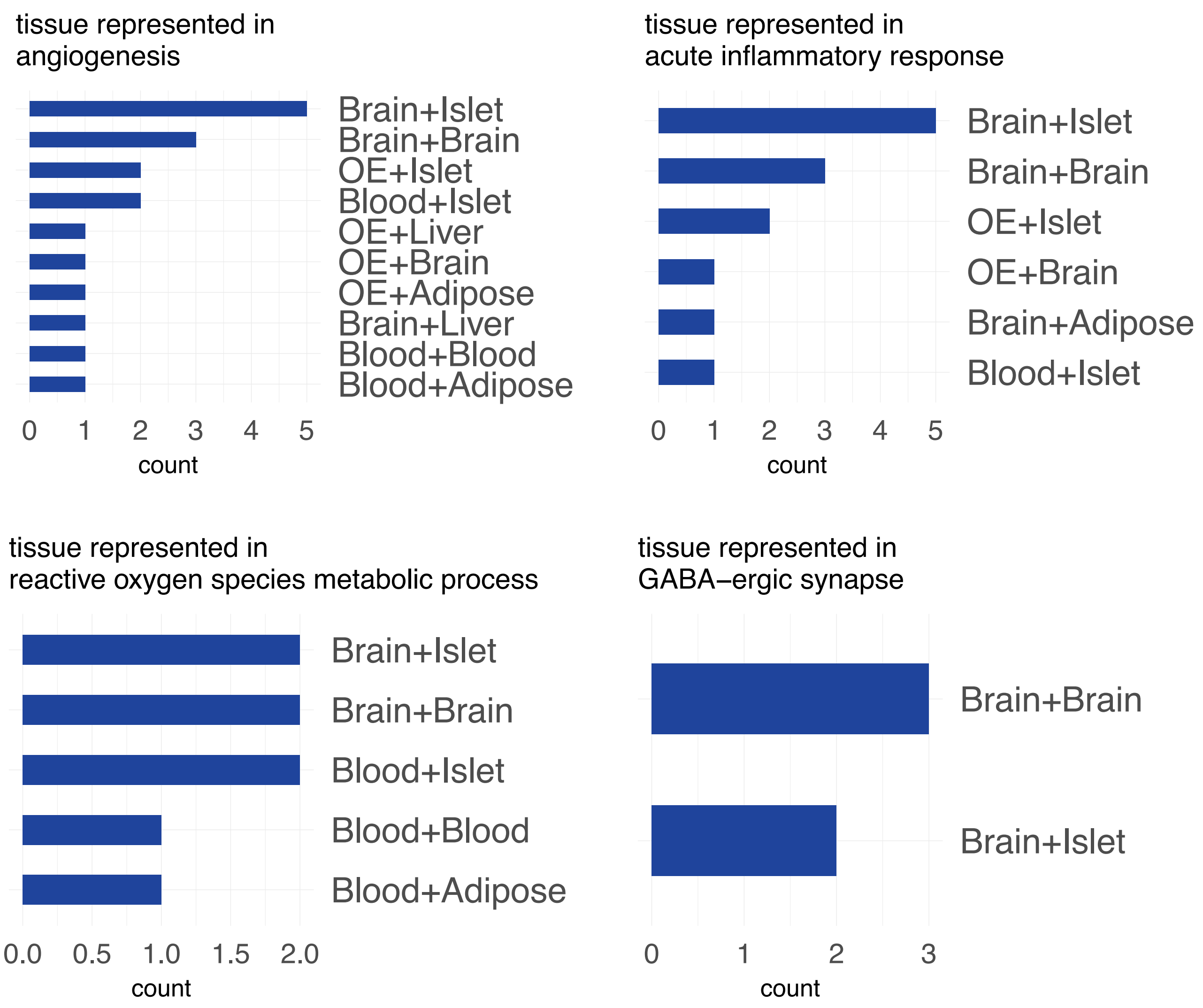

Supplementary Fig. 5 Tissue representation of the signature pairs enriched in different GO terms that corresponds to Fig. 4B

Supplementary Table S1. Summary of datasets used in analysis

	dataset	numsample	numcontrol	num case	condition	cohort	tissue	platform	reference
1	GSE21138	59	29	30	SCZ	PSY	Brain	GPL570[HG-U133_Plus_2] Affymetrix Human Genome U133 Plus 2.0 Array	https://www.ncbi.nlm.nih.gov/geo/query/acc.cgi?acc=GSE21138
2	GSE17612	49	22	27	SCZ	PSY	Brain	GPL570[HG-U133_Plus_2] Affymetrix Human Genome U133 Plus 2.0 Array	https://www.ncbi.nlm.nih.gov/geo/query/acc.cgi?acc=GSE17612
3	GSE27383	51	29	22	SCZ	PSY	Blood	GPL570[HG-U133_Plus_2] Affymetrix Human Genome U133 Plus 2.0 Array	https://www.ncbi.nlm.nih.gov/geo/query/acc.cgi?acc=GSE27383
4	GSE53987_2	70	18	52	BD	PSY	Brain	GPL570[HG-U133_Plus_2] Affymetrix Human Genome U133 Plus 2.0 Array	https://www.ncbi.nlm.nih.gov/geo/query/acc.cgi?acc=GSE53987
5	GSE53987_3	69	19	50	MDD	PSY	Brain	GPL570[HG-U133_Plus_2] Affymetrix Human Genome U133 Plus 2.0 Array	https://www.ncbi.nlm.nih.gov/geo/query/acc.cgi?acc=GSE53987
6	GSE74358	28	14	14	BD	PSY	Brain	GPL570[HG-U133_Plus_2] Affymetrix Human Genome U133 Plus 2.0 Array	https://www.ncbi.nlm.nih.gov/geo/query/acc.cgi?acc=GSE74358
7	GSE46449	88	39	49	BD	PSY	Blood	GPL570[HG-U133_Plus_2] Affymetrix Human Genome U133 Plus 2.0 Array	https://www.ncbi.nlm.nih.gov/geo/query/acc.cgi?acc=GSE46449
8	GSE73129	115	60	55	SCZ	PSY	OE	GPL570[HG-U133_Plus_2] Affymetrix Human Genome U133 Plus 2.0 Array	https://www.ncbi.nlm.nih.gov/geo/query/acc.cgi?acc=GSE73129
9	GSE7036	6	3	3	BD	PSY	Brain	GPL570[HG-U133_Plus_2] Affymetrix Human Genome U133 Plus 2.0 Array	https://www.ncbi.nlm.nih.gov/geo/query/acc.cgi?acc=GSE7036
10	GSE21935	42	19	23	SCZ	PSY	Brain	GPL570[HG-U133_Plus_2] Affymetrix Human Genome U133 Plus 2.0 Array	https://www.ncbi.nlm.nih.gov/geo/query/acc.cgi?acc=GSE21935
11	GSE76894	97	78	19	T2D	T2D	Islet	GPL570[HG-U133_Plus_2] Affymetrix Human Genome U133 Plus 2.0 Array	https://www.ncbi.nlm.nih.gov/geo/query/acc.cgi?acc=GSE76894
12	GSE76895	57	27	30	T2D	T2D	Islet	GPL570[HG-U133_Plus_2] Affymetrix Human Genome U133 Plus 2.0 Array	https://www.ncbi.nlm.nih.gov/geo/query/acc.cgi?acc=GSE76895
13	GSE23343	17	7	10	T2D	T2D	Liver	GPL570[HG-U133_Plus_2] Affymetrix Human Genome U133 Plus 2.0 Array	https://www.ncbi.nlm.nih.gov/geo/query/acc.cgi?acc=GSE23343
14	GSE15932	16	8	8	T2D	T2D	Blood	GPL570[HG-U133_Plus_2] Affymetrix Human Genome U133 Plus 2.0 Array	https://www.ncbi.nlm.nih.gov/geo/query/acc.cgi?acc=GSE15932
15	GSE71416	20	6	14	T2D	T2D	Adipose	GPL570[HG-U133_Plus_2] Affymetrix Human Genome U133 Plus 2.0 Array	https://www.ncbi.nlm.nih.gov/geo/query/acc.cgi?acc=GSE71416
16	GSE38396	8	4	4	T2D	T2D	Skin	GPL570[HG-U133_Plus_2] Affymetrix Human Genome U133 Plus 2.0 Array	https://www.ncbi.nlm.nih.gov/geo/query/acc.cgi?acc=GSE38396
17	GSE24752	6	3	3	HYP	CVD	Blood	GPL570[HG-U133_Plus_2] Affymetrix Human Genome U133 Plus 2.0 Array	https://www.ncbi.nlm.nih.gov/geo/query/acc.cgi?acc=GSE24752
18	GSE19339	8	4	4	ACS	CVD	Vessels	GPL570[HG-U133_Plus_2] Affymetrix Human Genome U133 Plus 2.0 Array	https://www.ncbi.nlm.nih.gov/geo/query/acc.cgi?acc=GSE19339
19	GSE48060	52	21	31	AMI	CVD	Blood	GPL570[HG-U133_Plus_2] Affymetrix Human Genome U133 Plus 2.0 Array	https://www.ncbi.nlm.nih.gov/geo/query/acc.cgi?acc=GSE48060
20	GSE66360	99	50	49	AMI	CVD	Vessels	GPL570[HG-U133_Plus_2] Affymetrix Human Genome U133 Plus 2.0 Array	https://www.ncbi.nlm.nih.gov/geo/query/acc.cgi?acc=GSE66360
21	GSE13985	10	5	5	ATH	CVD	Blood	GPL570[HG-U133_Plus_2] Affymetrix Human Genome U133 Plus 2.0 Array	https://www.ncbi.nlm.nih.gov/geo/query/acc.cgi?acc=GSE13985
22	GSE97320	6	3	3	AMI	CVD	Blood	GPL570[HG-U133_Plus_2] Affymetrix Human Genome U133 Plus 2.0 Array	https://www.ncbi.nlm.nih.gov/geo/query/acc.cgi?acc=GSE97320
23	GSE71226	6	3	3	CAD	CVD	Blood	GPL570[HG-U133_Plus_2] Affymetrix Human Genome U133 Plus 2.0 Array	https://www.ncbi.nlm.nih.gov/geo/query/acc.cgi?acc=GSE71226
24	GSE19303	48	8	40	CDM	CVD	Heart	GPL570[HG-U133_Plus_2] Affymetrix Human Genome U133 Plus 2.0 Array	https://www.ncbi.nlm.nih.gov/geo/query/acc.cgi?acc=GSE19303
25	GSE53987_1	66	18	48	SCZ	PSY	Brain	GPL570[HG-U133_Plus_2] Affymetrix Human Genome U133 Plus 2.0 Array	https://www.ncbi.nlm.nih.gov/geo/query/acc.cgi?acc=GSE53987
26	GSE161355	33	15	18	T2D	T2D	Brain	GPL570[HG-U133_Plus_2] Affymetrix Human Genome U133 Plus 2.0 Array	https://www.ncbi.nlm.nih.gov/geo/query/acc.cgi?acc=GSE161355
27	GSE27034	37	18	19	PAD	CVD	Blood	GPL570[HG-U133_Plus_2] Affymetrix Human Genome U133 Plus 2.0 Array	https://www.ncbi.nlm.nih.gov/geo/query/acc.cgi?acc=GSE27034

Supplementary Table S2. Summary of demographic and clinical information

label	levels	bipolar disorder	control	cvd	major depressive disorder	schizophrenia	t2d
gender	female	23 (22.8)	136 (34.6)	13 (27.1)	22 (44.0)	63 (30.9)	33 (38.8)
	male	78 (77.2)	257 (65.4)	35 (72.9)	28 (56.0)	141 (69.1)	52 (61.2)
tissue	associative striatum	17 (16.8)	18 (5.4)		16 (32.0)	18 (8.8)	
	ba46	17 (16.8)	29 (8.7)		17 (34.0)	45 (22.0)	
	hippocampus	18 (17.8)	18 (5.4)		17 (34.0)	15 (7.3)	
	leukocytes from whole blood	49 (48.5)	39 (11.7)				
	ba10		22 (6.6)			27 (13.2)	
	ba22		19 (5.7)			23 (11.2)	
	blood		30 (9.0)	40 (58.8)			
	laser captured cells from human temporal cortex		15 (4.5)				18 (64.3)
	liver		7 (2.1)				10 (35.7)
	lymphoblast from blood		41 (12.3)			36 (17.6)	
	oe		19 (5.7)			19 (9.3)	
	pbmc		47 (14.2)	19 (27.9)		22 (10.7)	
	peripheral blood		9 (2.7)	9 (13.2)			
	pre-frontal cortex (ba46)		19 (5.7)				
age	Mean (SD)	43.4 (12.2)	50.9 (19.1)	49.2 (10.2)	45.6 (10.3)	50.7 (19.4)	68.3 (10.9)

Supplementary Table S3. Dopaminergic systems related terms enriched in scz-t2d signature pairs

	ID	pathway	pvalue	p.adjust	qvalue	idx	ConditionClass	CondtionType	CohortClass	CohortType
1	GO:0042053	regulation of dopamine metabolic process	0,004911423	0,044140039	0,034983667	GSE17612_R5_S4+GSE76895_R19_S1	SCZ-T2D	inter-class comorbid	PSY-T2D	comorbid
2	GO:1904948	midbrain dopaminergic neuron differentiatio	0,003493951	0,086663046	0,075687949	GSE27383_R12_S1+GSE71416_R5_S2	SCZ-T2D	inter-class comorbid	PSY-T2D	comorbid
3	GO:1903350	response to dopamine	0,010069536	0,11202886	0,096326947	GSE73129_R17_S9+GSE161355_R9_S1	SCZ-T2D	inter-class comorbid	PSY-T2D	comorbid
4	GO:1903351	cellular response to dopamine	0,010069536	0,11202886	0,096326947	GSE73129_R17_S9+GSE161355_R9_S1	SCZ-T2D	inter-class comorbid	PSY-T2D	comorbid
5	GO:0042416	dopamine biosynthetic process	7,25718E-05	0,022096203	0,01962034	GSE73129_R19_S1+GSE71416_R16_S3	SCZ-T2D	inter-class comorbid	PSY-T2D	comorbid
6	GO:0042417	dopamine metabolic process	0,002309607	0,09290778	0,082497532	GSE73129_R19_S1+GSE71416_R16_S3	SCZ-T2D	inter-class comorbid	PSY-T2D	comorbid
7	GO:0071542	dopaminergic neuron differentiation	0,00335158	0,090035546	0,080385695	GSE53987_1_R11_S3+GSE71416_R16_S3	SCZ-T2D	inter-class comorbid	PSY-T2D	comorbid
8	GO:0014046	dopamine secretion	0,000183476	0,036218198	0,030920565	GSE53987_1_R8_S5+GSE161355_R6_S5	SCZ-T2D	inter-class comorbid	PSY-T2D	comorbid
9	GO:0014059	regulation of dopamine secretion	0,000183476	0,036218198	0,030920565	GSE53987_1_R8_S5+GSE161355_R6_S5	SCZ-T2D	inter-class comorbid	PSY-T2D	comorbid
10	GO:0015872	dopamine transport	0,000558922	0,068956951	0,058870623	GSE53987_1_R8_S5+GSE161355_R6_S5	SCZ-T2D	inter-class comorbid	PSY-T2D	comorbid

Supplementary Table S4. Serotonergic systems related terms enriched in CVD-MDD signature pairs

	ID	pathway	pvalue	p.adjust	qvalue	idx	ConditionClass	CondtionType	CohortClass	CohortType
1	GO:0004993	G protein-coupled serotonin receptor activity	0,005456162	0,107188776	0,091997673	GSE19339_R7_S1+GSE53987_3_R15_S5	ACS-MDD	inter-class comorbid	CVD-MDD	comorbid
2	GO:0098664	G protein-coupled serotonin receptor signaling pathway	0,005456162	0,107188776	0,091997673	GSE19339_R7_S1+GSE53987_3_R15_S5	ACS-MDD	inter-class comorbid	CVD-MDD	comorbid
3	GO:0099589	serotonin receptor activity	0,005456162	0,107188776	0,091997673	GSE19339_R7_S1+GSE53987_3_R15_S5	ACS-MDD	inter-class comorbid	CVD-MDD	comorbid
4	GO:0007210	serotonin receptor signaling pathway	0,006816027	0,115979792	0,099542801	GSE19339_R7_S1+GSE53987_3_R15_S5	ACS-MDD	inter-class comorbid	CVD-MDD	comorbid
5	GO:0001820	serotonin secretion	0,001076566	0,02054598	0,016719899	GSE66360_R14_S8+GSE53987_3_R15_S13	AMI-MDD	inter-class comorbid	CVD-MDD	comorbid
6	GO:0006837	serotonin transport	0,004400874	0,047194137	0,038405624	GSE66360_R14_S8+GSE53987_3_R15_S13	AMI-MDD	inter-class comorbid	CVD-MDD	comorbid
